# Liver function monitoring: a prospective nested case-control study of *Salvia miltiorrhiza* polyphenol injection

**DOI:** 10.1038/s41598-020-60608-z

**Published:** 2020-02-26

**Authors:** Jin-quan Cheng, Qing-ping Shi, Feng Ding, Ling-ti Kong, Mei-ling Yu, Can Wang

**Affiliations:** 1 Department of Pharmacy, the First Affiliated Hospital of Bengbu Medical College, Bengbu, Anhui China; 2 School of Pharmacy, Bengbu Medical College, Bengbu, Anhui China

**Keywords:** Adaptive clinical trial, Adaptive clinical trial, Adaptive clinical trial, Risk factors, Risk factors

## Abstract

Instructions for *Salvia miltiorrhiza* polyphenol injections indicate abnormal liver function as an occasional adverse reaction, but the incidence of this adverse drug reaction (ADR) has increased in recent years. We assessed *S*. *miltiorrhiza* polyphenol ADRs by performing a nested case-control study(NCCS) and meta-analysis. In the NCCS, 2633 patients receiving this treatment in the First Affiliated Hospital of Bengbu Medical College were enrolled. Logistic regression models found that in 58 (2.2%) patients experiencing abnormal liver function, the risk for liver dysfunction was associated with sulfa drug allergy (OR = 7.874, 95%CI (1.280, 48.447), P = 0.026), payment methods (OR = 0.106, 95%CI (0.012, 0.934), P = 0.043), duration of administration (OR = 0.922, 95%CI (0.862, 0.986), P = 0.017), cefathiamidine (OR = 0.441, 95%CI (0.216, 0.900), P = 0.025), human serum albumin (OR = 1.958, 95%CI (1.011, 3.789), P = 0.046), Dazhu Rhodiola injection (OR = 2.599, 95%CI (1.112, 6.070), P = 0.027), or reduced glutathione (OR = 0.394, 95%CI (0.188, 0.826), P = 0.014). Meta-analysis of reports on *S*. *miltiorrhiza* polyphenol ADRs in controlled trials and other observational studies included 676 patients, of which 17 (2.17%; 95%CI (0.0105, 0.0358)) presented with liver dysfunction; associated ADR risk factors included co-administration of other drugs. Our NCCS and meta-analysis had similar ADR incidence rates, which were higher than the rate in the drug instructions. This study provides guidance for assessing liver dysfunction risks associated with *S*. *miltiorrhiza* polyphenol injections.

## Introduction

**Figure 1:**
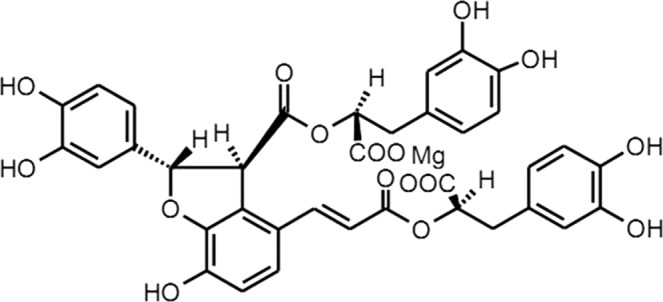
Chemical structure of *Salvia miltiorrhiza* magnesium lithospermate B.

An injectable formulation of *Salvia miltiorrhiza* polyphenols prepared as a multi-effect antioxidant freeze-dried powder was jointly developed by the Shanghai Institute of Materia Medica, Chinese Academy of Sciences, and Shanghai Green Valley (Group) Co., Ltd. Its main active component is magnesium lithospermate B (Fig. 1), a salvianolic acid B salt extracted from the traditional Chinese medicinal herb Danshen *(S*. *miltiorrhiza)*. The injectable formulation, which exerts anti-arrhythmic and antioxidant effects and protects the endothelium, is used to treat coronary heart disease, angina pectoris, and ischemic stroke^1–7^.

The abnormal liver function indicated in the *Salvia miltiorrhiza* polyphenols injection instructions is an occasional adverse reaction. It is clinically manifested mainly as elevated glutamine-pyruvate transaminase activity, which can be improved after drug withdrawal. This is based on early research conducted under normal and reasonable use. The probability of occurrence of occasional adverse reactions ranges from 0.1% to 1% according to the Council for International Organizations of Medical Sciences (CIOMS)^8^. However, with the increasing use of *S*. *miltiorrhiza* polyphenol injections, especially when used in combination with other drugs, the clinical manifestation and severity of liver dysfunction have far exceeded the ones known during their launch^9^. Case reports, randomized placebo-controlled clinical studies, and literature analyses describe abnormal glutamic-pyruvic transaminase, alanine aminotransferase, and total bilirubin adverse reactions detected by liver function monitoring during treatment^10–15^. However, the incidence, outcome, and severity of the adverse reactions were not systematically analysed.

Therefore, we conducted both a NCCS and an meta-analysis to investigate the influencing factors and incidence of adverse reactions to *S*. *miltiorrhiza* polyphenol injection. We aimed to develop an approach for reducing adverse reactions to *S*. *miltiorrhiza* polyphenols, methods for its safety risk assessment, and directions for future research.

## Methods

### Study design

This NCCS protocol was approved by the First Affiliated Hospital of Bengbu Medical College and the Research Institute of the State Administration of Traditional Chinese Medicine. This study was carried out in accordance with an invention patent for a method and system of re-evaluating drugs after market launch. The research protocol had been used for the safety re-evaluation of other drugs and had previously been published elsewhere. In addition, CHPS software had obtained the copyright from the National Copyright Administration of the People’s Republic of China.

#### Nested case-control study(NCCS) design

**Figure 2:**
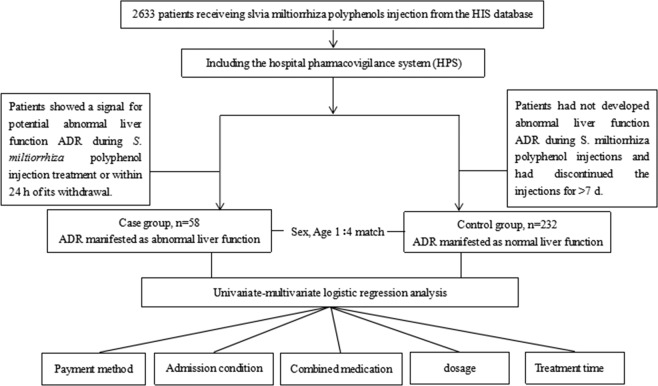
Flowchart of inpatient selection and processing.

We performed a prospective, large-sample nested case-control study (NCCS) based on liver function monitoring of *S*. *miltiorrhiza* polyphenol injection. From June 1st, 2017 to December 31st, 2018, patients receiving *S*. *miltiorrhiza* polyphenol injection in the First Affiliated Hospital of Bengbu Medical College were automatically included the study via the hospital pharmacovigilance system (HPS). We set the alert threshold for liver function abnormality in the HPS. If the value of a patient’s liver function triggers an alert signal, the patient’s liver function abnormality is automatically assigned into the case group. We used the patient’s age and gender as the matching conditions, and the control group was determined according to the principle of NCCS design in the remaining untriggered patient population. The design of the NCCS is shown in Fig. 2.

#### Alert thresholds for abnormal liver function

The HPS, which collects real-world clinical data, is an information system established to support sentinel surveillance, record and evaluate ADRs, conduct drug monitoring and re-evaluation, and obtain drug warning information^16–18^. The following ADR alert thresholds for abnormal liver function during *S*. *miltiorrhiza* polyphenol treatment or within 24 h of its withdrawal were used as settings in the HPS: glutamic-pyruvic transaminase or glutamic oxaloacetic transaminase values above 40 U/L or alkaline phosphatase values exceeding the normal range of 40–110 U/L; the total bilirubin exceeding the normal range of 1.71–17.1 μmol/L, the indirect bilirubin exceeding the normal range of 1.7–13.7 μmol/L, or the direct bilirubin exceeding the normal range of 1.71–7 μmol/L; the total serum protein, serum albumin, or serum globulin values exceeding the normal range of 60–80 g/L, 40–55 g/L, or 20–30 g/L, respectively; the prothrombin time, cholinesterase, and total serum bile acid values exceeding the normal range of 12–14 s, 130–310 U/L, and 10 μmol/L, respectively. Patients who triggered the alert during medication were categorized as suspected abnormal liver function. The HPS contained basic information about medications administered to all inpatients during hospitalization.

#### Case and control group

The case group included all patients who triggered a alert signal in the HPS to indicate abnormal liver function. Patients were matched for age (±5 years age difference) and sex. The patients who were randomly selected from the remaining untriggered signals were assigned to the control group. The ratio of the case to the control group was 1:4. In addition, the causal relationship between *S*. *miltiorrhiza* polyphenol injection and abnormal liver function ADR was evaluated according to the Naranjo evaluation scale^19,20^. A patient’s score of >5 points indicated a significant relationship. The specific grouping situation was as follows:

Case group. A total of 58 patients were included based on a signal for potential abnormal liver function ADR during *S*. *miltiorrhiza* polyphenol injection treatment or within 24 h of its withdrawal.

Control group. A total of 232 control group patients were randomly selected using the case-to-control ratio of 1:4. Patients had not developed abnormal liver function ADR during *S*. *miltiorrhiza* polyphenol injections and had discontinued the injections for >7 d.

#### Meta-analysis design

Inclusion criteria. (1) Randomized controlled trials (RCTs) and other observational studies written in Chinese or English were included. (2) The subjects were patients who received *S*. *miltiorrhiza* polyphenol treatment, regardless of age, sex, or disease type. (3) The test drug was the *S*. *miltiorrhiza* polyphenol injection, administered as an intravenous drip and used alone or in combination with other drugs. 4) Outcome measures reported the incidence and rate of abnormal liver function.

Exclusion criteria. (1) The description of the intervention involving drugs was unclear, and the ADR could not be evaluated. 2) ADRs were not previously described. (3) The data was incomplete and could not be analysed. (4) Short reports. (5) Reviews. (6) Animal or cell experiments. (7) Basic medical research.

We used the Cochrane System Evaluator Manual v. 5.1.0 offset risk assessment tool^21^ and MOOSE guidelines^22^ to assess the quality of the RCTs and observations. Each of seven quality criteria was counted as one point, and a total score of ≥4 was indicative of high-quality literature^23^. Quality score evaluation and basic data extraction from the literature were conducted independently by two evaluators, and their results were cross-checked for accuracy. When an inconsistency occurred, the research team determined whether to include the study or not.

### Data source

NCCS data were obtained from the medical records in the Hospital Information System (HIS) of the First Affiliated Hospital of Bengbu Medical College. Meta-analysis data were obtained from PubMed, Embase, the Cochrane Library, Web of Science, Clinical Trials, CNKI, VIP, and Wanfang Data. The retrieval time to build our database lasted until June 15th, 2019. The search was performed by combining the subject and the free words, then, the results were screened according to the inclusion and exclusion criteria. The PubMed search strategy was as follows:

“*Salvia miltiorrhiza* polyphenols for injection” [Title/Abstract] OR “Dan Shen Duo Fen Suan Yan” [Title/Abstract]
“Coronary heart disease” [Title/Abstract] OR “Angina pectoris” [Title/Abstract] OR “Myocardial infarction” [Title/Abstract] OR “Cerebral infarction” [Title/Abstract] OR “Thrombosis” [Title/Abstract] OR “Adverse drug reaction” [Title/Abstract]
(1) AND (2)

### Data extraction

NCCS data were extracted by a professional data expert. The data included patient’s sex, age, drug allergy history, admission condition, payment method, comorbidities, combined medication, and drug information (including specifications, usage and dosage, solvent, duration of administration). The data of 2633 patients who received *S*. *miltiorrhiza* polyphenol injections were uniformly standardized before analysis^24^. Meta-analysis data included author names, study title, publication date, study type, observation object, intervention measures, and quality evaluation scores. Possible offsets were avoided during data extraction, but if unavoidable, a sensitivity analysis was performed.

### Statistical analyses

NCCS data were analysed by χ^2^ test, Wilcoxon test, and univariate and multivariate conditional logistic regression analyses. Abnormal liver function ADR was the dependent variable, while possible causes like admission condition, allergy history, payment method, comorbidities, solvent type, dosage, duration of administration, and top 43 frequently combined drugs were independent variables(Supplementary Table 1). Statistical analysis was performed using SPSS V21.0 statistical software. Meta-analysis data were processed using R3.5.0 software. The confidence interval for each effect variable was expressed as 95% CI. Homogeneity test (Q test) was used to evaluate heterogeneity. The test level was α = 0.1, and the size of heterogeneity was quantitatively determined by combining *I*^2^ (judgment criterion *P* ≥ 0.1 or *I*^2^ ≤ 50%)^25,26^. Heterogeneity was considered significant when *p* < 0.1 and *I*^2^ > 50%. When *P* ≥ 0.1 and *I*^2^ ≤ 50%, the fixed-effect model was used for the meta-analysis; otherwise, a random-effect model was used.

### Ethics approval

Ethics approval was obtained from the Clinical Medicine Research Ethics Committee of the First Affiliated Hospital of Bengbu Medical College. The patient data used in this study were approved by the Ethics Committee of the First Affiliated Hospital of Bengbu Medical College. Before enrolment, each patient was full explained about the study and signed an informed consent form.

## Results

### Nested case-control study results

#### Demographic characteristics

The NCCS included a total of 2633 patients who received *S*. *miltiorrhiza* polyphenol injection according to their medical records. During the study, 58 patients who triggered an alert singal during treatment had a Nangjo score of more than 5 points. Thus, the incidence rate of abnormal liver function was 2.2% (58/2633). Among the 58 patients were 30 patients with increased glutamic aminotransferase, 19 with increased aspartate aminotransferase, 5 with increased total bilirubin, and 4 with other ADRs related to liver function. Fifty patients with general ADRs improved after discontinuation. Eight patients were cured after treatment with reducible glutathione and other prophylactin.

Based on the NCCS design, 290 patients were included in the study. The case group had 58 patients with an average age of 64.12 ± 16.28 years, and the control group had 232 patients with an average age of 64.41 ± 16.86 years. Patients were 16–94 years of age. The Wilcoxon test did not indicate significant age differences between the groups (P  =  0.745). The sex ratio was similar within each group and did not significantly differ between both groups (P = 0.594). Thus, age and sex distribution were similar in both groups (Table 1).

**Table 1:** Distribution of inpatients by age and sex.

Groups	*n*	Age, *n* (%)				Sex, *n* (%)	
		≤40 years	41–60 years	61–80 years	>80 years	Male	Female
Case group	58	4 (9.52%)	15 (33.33%)	30 (28.57%)	9 (28.57%)	31 (53.45%)	27 (46.55%)
Control group	232	24 (10.34%)	64 (27.59%)	99 (42.67%)	45 (19.40%)	133 (57.33%)	99 (42.67%)

#### Factors affecting abnormal liver function

Univariate conditional logistic regression analysis showed that sulfa drug allergy, admission condition, payment methods, duration of administration, hypertension and combined use with 10% potassium chloride, cefathiamidine, human serum albumin, Dazhu Rhodiola injection, ambroxol oral solution, reduced glutathione, mannitol injection, and vitamin B6 are associated with ADRs of *S*. *miltiorrhiza* polyphenol injections (P < 0.05) (Table 2). These statistically significant factors were used as candidate variables to establish a multivariate conditional logistic regression model. The experimental variables were gradually screened backward using P < 0.05 as test level. The results showed sulfa drug allergy, payment methods, duration of administration, and combined use with cefotaxime, human serum albumin, Dazhu Rhodiola injection, and reduced glutathione were statistically significant risk factors (Table 3).

**Table 2:** Univariate conditional logistic regression analysis.

Variables	Univariate conditional logistic regression analysis				
	Regression coefficient (B)	Standard error (SE)	Wald χ^2^	OR (95%CI)	*P*
Sulfa drug allergy	1.942	0.619	9.834	6.971 (2.071, 23.463)	0.002
Payment methods	−2.401	0.802	8.961	0.091 (0.019, 0.437)	0.003
Admission condition	−1.206	0.532	5.140	0.299 (0.106, 0.849)	0.023
Duration of administration (d)	−0.049	0.024	4.224	0.952 (0.908, 0.998)	0.040
Hypertension	−1.864	0.857	4.735	0.155 (0.029, 0.831)	0.030
10% potassium chloride	−1.325	0.618	4.594	0.266 (0.079, 0.893)	0.032
Cefathiamidine	−0.706	0.277	6.498	0.494 (0.287, 0.850)	0.011
Human serum albumin	0.842	0.282	8.939	2.320 (1.336, 4.029)	0.003
Dazhu rhodiola injection	0.670	0.324	4.293	1.955 (1.037, 3.685)	0.038
Reduced glutathione	−0.863	0.302	8.182	0.422 (0.233, 0.762)	0.004
Ambroxol oral solution	−1.608	0.713	5.084	0.200 (0.049, 0.810)	0.024
Mannitol injection	−5.162	2.563	4.057	0.006 (0.000, 0.087)	0.044
Vitamin B6	−1.884	0.842	5.003	0.152 (0.029, 0.792)	0.025

**Table 3:** Multivariate conditional logistic regression analysis.

Risk factors	Multivariate conditional logistic regression analysis				
	Regression coefficient (B)	Standard error (SE)	Wald χ^2^	OR (95%CI)	*P*
Sulfa drug allergy	2.064	0.927	4.956	7.874 (1.280, 48.447)	0.026
Payment method	−2.242	1.109	4.085	0.106 (0.012, 0.934)	0.043
Duration of administration (d)	−0.081	0.034	5.674	0.922 (0.862, 0.986)	0.017
Cefathiamidine	−0.819	0.364	5.056	0.441 (0.216, 0.900)	0.025
Human serum albumin	0.672	0.337	3.972	1.958 (1.011, 3.789)	0.046
Dazhu rhodiola injection	0.955	0.433	4.866	2.599 (1.112, 6.070)	0.027
Reduced glutathione	−0.932	0.378	6.074	0.394 (0.188, 0.826)	0.014

### Meta-analysis results

#### Literature search

Our literature search identified 3109 relevant studies. Chinese databases contributed 3051 studies, including 899 from VIP, 1066 from Wanfang Data, and 1086 from the China National Knowledge Infrastructure. Fifty-eight studies were retrieved from English databases, including 7 from the Cochrane Library, 51 from PubMed, but 0 from other databases. All studies were screened based on the inclusion and exclusion criteria, yielding 9 studies for the meta-analysis, including 3 RCTs, 4 non-RCTs, and 2 observational studies.

#### Basic research information

A total of 676 patients from 9 studies were selected, including 676 patients who received *S*. *miltiorrhiza* polyphenol injections. In 17 patients who presented with abnormal liver function, the main manifestations were alanine aminotransferase, aspartate aminotransferase, and total bilirubin elevation. A single daily dose contained 100–200 mg/d, using 5% glucose or 0.9% sodium chloride as solvents. Main indications for treatment were cardiovascular and cerebrovascular diseases. Eight articles had a quality score greater than or equal to 3, which was the threshold score for a high-quality study. The basic information derived from these articles is summarized (Table 4).

**Table 4:** Basic information from the literature used in the meta-analysis.

Study name	Type	Indications	Age (years)	Observation group	Control group	Total sample size	*Salvia miltiorrhiza* injection					Adverse reaction manifestation	Score
				Total (Male/Female)	Total (Male/Female)		Solvent	Dosage (mg · d^−1^)	Time (*d*)	Method	Combination therapy		
Wang S 2014	1	SA	45–86	30 (15/15)	15 (8/7)	45	2	200	10	1	—	1 patient presented mild elevation of ALT	4
Hong YQ 2016	1	CHDA	62.30 ± 2.50	94 (63/31)	94 (62/32)	188	1	200	14	1	—	1 patient presented elevated ALT	3
Luo XD 2017	1	ACI	54.9 ± 13.6	35 (19/16)	35 (23/12)	70	2	200	14	2	—	1 patient showed mild elevation of ALT and AST	4
Wang X 2010	2	SA	66.98±9.09	50 (29/21)	38 (21/17)	88	1	200	14	1	—	2 patients presented mild elevation of ALT	4
Tian Y 2019	2	IS	56.74 ± 7.41	61 (33/28)	61 (35/26)	122	2	200	14	2	Edaravone	1 patient presented elevated TBIL	4
Liu WW 2018	2	ST	18–70	33	32	65	—	100	56	1	—	2 patients presented abnormal liver function	3
Fang G 2017	2	IS	57.83 ± 7.79	75 (32/43)	75 (34/41)	150	2	100	14	1	—	2 patients presented liver damage	4
Huang ZE 2019	3	ACI	70.97 ± 12.27	87 (46/41)	—	87	2	100–200	≤14	—	—	1 patient presented AST abnormalities and 1 presented hepatic hemorrhage	3
Xiang KL 2016	3	SA	60–82	211	—	211	1	200	10–14	2	Nitroglycerin	5 patient presented elevated ALT	2

Note:

Abbreviations: SA, stable angina; CHDA, coronary heart disease angina; ACI, acute cerebral infarction; IS, ischemic stroke; ST, smear tuberculosis; ALT, alanine aminotransferase; AST, aspartate aminotransferase; TBIL, total bilirubin. Types of study: 1 indicates RCT, 2 indicates non-randomised controlled studies, 3 indicates other observational studies. Mode of administration: 1 indicates single use; 2 indicates combined use. Solvent type: 1 indicates 5% glucose solution; 2 indicates 0.9% sodium chloride solution. —: not provided.

### Incidence of abnormal liver function determined by meta-analysis

**Figure 3:**
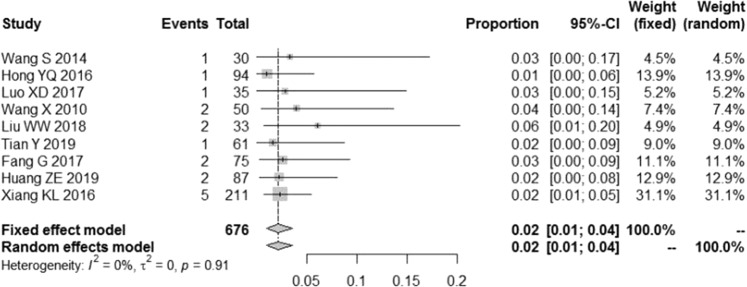
Forest plot to assess incident distribution in meta-analysis.

Among 676 patients receiving *S*. *miltiorrhiza* polyphenol injections, 17 exhibited abnormal liver function. Thus, the incidence rate of abnormal liver function in the treatment group was 2.17% by meta-analysis. A forest plot for abnormal liver function in our meta-analysis was created (Fig. 3).

### Publishing bias and sensitivity analysis

**Figure 4:**
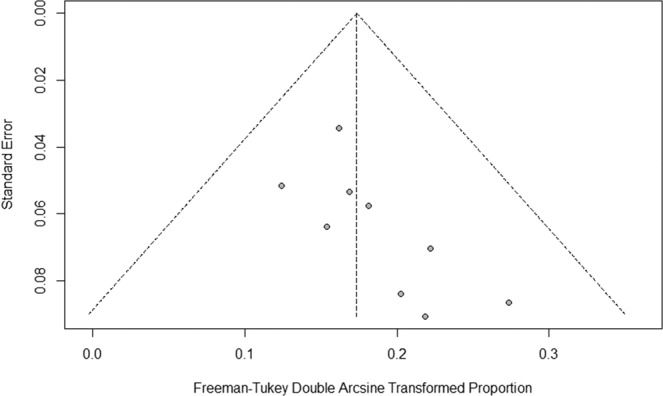
Funnel plot to assess publication bias in meta-analysis.

We used a funnel plot method to evaluate potential publication bias because the number of studies was too small for performing the Egger regression or Begg correlation test. There was no symmetric distribution of the data, probably due to the small study number (Fig. 4). The meta-analysis was performed after removing literature items showing poor quality and high specificity. However, there was no significant change in the incidence rate of abnormal liver function, indicating that the meta-analysis generated a robust result.

### Comparison of the ADR incidence rates derived from the NCCS and meta-analysis

We performed a χ2 test on the incidence rate of liver dysfunction in patients treated with *S*. *miltiorrhiza* injection using the patient data from the NCCS and the meta-analysis. There was no statistically significant difference in the ADR incidence rates of *S*. *miltiorrhiza* treatment between the NCCS data on liver dysfunction and the meta-analysis (P = 0.627).

## Discussion

Post-marketing clinical research on drugs is mainly focused on re-evaluating their safety and effectiveness. RCTs are the gold standard for drug efficacy studies, but there are shortcomings in re-evaluating post-marketing drug safety^27^. Recently, satisfactory results were obtained by applying NCCS and meta-analysis in safety re-evaluation studies of Chinese medicine injections^23,28,29^. An NCCS requires large-sample clinical data and uses broad inclusion criteria and epidemiological research methods to assess the clinical application of drugs or other interventions (including diagnosis, treatment, and prognosis) in a truly unbiased or less biased population^30^. An NCCS has one cohort of all disease cases that meet specific inclusion criteria; typically, the disease onset occurred within a certain period. Further criteria are applied within the cohort to establish one or more controls along with the treatment group. The groups are matched based on factors such as age, sex, address, and/or ethnicity, and the relevant patient information is subjected to statistical analyses^31^. An NCCS is often used for safety risk assessments^32–34^.

The NCCS design is economical and has the advantage that it avoids potential selective and recall biases of classic case-control studies incurred when collecting data. It reduces the interference of confounding factors by relying on comparable, well-balanced case and control groups, which allows a good extrapolation of study results.

Meta-analysis requires extensive RCTs and other observational studies, which prevents selective migration, ensures comparability between groups, generates highly authentic results, and has a high evidence level. A meta-analysis of widely used RCT research data enables objective evaluation of the evidence and a more accurate and objective assessment of effect indicators.

The innovation of this study is that it evaluated the incidence rate of abnormal liver function for *S*. *miltiorrhiza* polyphenol injections based on a meta-analysis and a logistic regression analysis of real-world data. This approach provides a new method that can be used for further studies on the adverse effects of *S*. *miltiorrhiza* polyphenol injections. Both NCCS and meta-analysis can be used as a basis for prospective clinical studies. The methods complement each other and generate comparable results. They can innovatively evaluate the safety of *S*. *miltiorrhiza* polyphenol injections in clinical practice, providing an evidence-based and rigorous reference for clinicians.

With the wide clinical application of *S*. *miltiorrhiza* polyphenol injections, the adverse reaction reports have gradually increased^10,35–37^. The NCCS determined an incidence rate of 2.2% for abnormal liver function adverse reactions to *S*. *miltiorrhiza* polyphenol injections, and the meta-analysis derived the incidence rate of 2.17%. There was no significant difference in the incidence rate of abnormal liver function adverse reactions between our two studies, although the two methods were quite different. The abnormal liver function described in the *S*. *miltiorrhiza* polyphenols injection for instructions is an occasional adverse reaction with a probability range form 0.1% to 1%^8^. The clinical manifestations described in the drug instructions were similar to those identified in our two studies, although the corresponding incidence rate was much lower than in our studies. This may be due to the limited sample size of studies conducted before the drug was approved for the market, and the clinical irrational use of drugs after the market had led to an increase in the incidence of abnormal liver function. Moreover, the subjects in the earlier studies were also strictly screened, while our study included all patients who received *S*. *miltiorrhiza* polyphenols within the same period, and our inclusion criteria were broader. There were 9 earlier studies, including 4 non-randomized controlled studies, 2 observational studies, and 3 randomized controlled studies, which had also relatively broad inclusion criteria. In our NCCS and meta-analysis, we found that the abnormal liver function adverse reactions to *S*. *miltiorrhiza* polyphenols were closely associated with the combined use of drugs. We know that due to the complex condition of patients, the effect of single-agent treatment is often poor, and treatment with combination therapy is very common in modern medicine. However, the study of the combination therapy before market launch is strictly limited.

Previous studies have shown that factors affecting ADRs of *S*. *miltiorrhiza* polyphenol injections might be age, sex, solvent, and concurrent medications^13–15,38–42^. In this study, univariate and multivariate conditional logistic regression analysis showed that the sulfa drug allergy, payment methods, and duration of administration were also risk factors for abnormal liver function associated with *S*. *miltiorrhiza* polyphenol injections. Moreover, administration of *S*. *miltiorrhiza* polyphenol injections in combination with cefathiamidine, human serum albumin, Dazhu Rhodiola injection, or reduced glutathione increased the risk of abnormal liver function ADRs.

In previous studies, Lv *et al*.^41^ reported that factors affecting ADRs of *S*. *miltiorrhiza* polyphenol injections were age, sex, and concurrent medications. Liao *et al*.^42^ reported that admission condition, solvent, and concurrent medications were risk factors for allergic reactions to *S*. *miltiorrhiza* polyphenols. However, these studies did not focus on the risk factors associated with liver dysfunction caused by *S*. *miltiorrhiza* polyphenol injections. We identified sulfa drug allergy, payment methods, duration of administration, and concurrent medications as risk factors for abnormal liver function ADRs associated with *S*. *miltiorrhiza* polyphenol treatment. Our results varied from earlier reports^41,42^. Our case data source, the range of confounding factors, and the research methods differed from those in previous studies^41,42^. This study was an NCCS and meta-analysis of re-evaluating the safety of liver function of *S*. *miltiorrhiza* polyphenol injections, which included a broader analysis of confounding factors.

Since *S*. *miltiorrhiza* polyphenol injections are covered by the national medical insurance in China, the patient can pay the treatment through medical insurance, thereby reducing the economic pressure. Because of this, clinicians may be more inclined to prescribe *S*. *miltiorrhiza* polyphenol injections. However, frequent use of the same drug may increase the risk of ADRs. Therefore, this study used the payment method as an independent variable for logistic regression analysis and concluded that payment method was a risk factor for abnormal liver function adverse reactions to *S*. *miltiorrhiza* polyphenol injections. This may be a new finding that complements the results of previous studies and provides a new reference for clinical use.

This study combined NCCS methods and meta-analysis to generate a more scientific and reliable reference for the rational clinical use of *S*. *miltiorrhiza* polyphenol injections and the prevention and control of adverse reactions. Although all clinical data in this study were derived from the HIS database, our results were more robust, evidence-based, and extrapolated, compared with those of previous studies^13–15,42,43^. Due to limited data availability, it was impossible to make a comprehensive judgment on symptoms and clinical manifestation in patients. In future studies, it will be necessary to increase the sample size, expand the scope of the cases, and establish a multi-centre, large-sample study to obtain more reliable results.

In conclusion, the incidence rate of abnormal liver function adverse reactions to *S*. *miltiorrhiza* polyphenols was approximately 2.0%, which was higher than the value indicated in the drug instruction. This study showed that the risk factors for abnormal liver function adverse reactions to *S*. *miltiorrhiza* polyphenol injections were sulfa drug allergy, payment methods, duration of administration, along with co-administration of cefathiamidine, human serum albumin, Dazhu Rhodiola injection, and reduced glutathione. Therefore, when *S*. *miltiorrhiza* polyphenol injections are used in a clinical setting, attention should be paid to the indications of the medication, drug allergy history, payment methods and duration of administration. Especially, when combined with other drugs, preventive measures should be taken to limit the risk of compatibility changes. Clinicians can minimise the risk of adverse reactions associated with frequent use by intermittently prescribing other drugs with the same indication.

## Supplementary information

Dataset 1.

## Data Availability

The datasets generated and analysed during the current study are available from the corresponding author on reasonable request. The current study of trial registration number from the Chinese Clinical Trial Registration Centre is ChiCTR1900024340.

## References

[1] Han B (2011). Protective effects of salvianolate on microvascular flow in a porcine model of myocardial ischaemia and reperfusion. Arch. Cardiovasc. Dis..

[2] Qi Y (2018). Patient characteristics associated with treatment response in patients receiving salvianolate injection for stable angina. J. Evid. Based Med..

[3] Dong P (2018). Cost-consequence analysis of salvianolate injection for the treatment of coronary heart disease. Chin. Med..

[4] NanZhu Y, AiChun J, Xin L (2018). Salvianolate injection in the treatment of acute cerebral infarction: A systematic review and a meta-analysis. Med..

[5] Fish JM (2006). Dimethyl Lithospermate B, an Extract of Danshen,Suppresses Arrhythmogenesis Associated With the Brugada Syndrome. Circulation.

[6] Kim SH (2010). Natural therapeutic magnesium lithospermate B potently protects the endothelium from hyperglycaemia-induced dysfunction. Cardiovasc. Res..

[7] Qu J (2011). The protective effect of magnesium lithospermate B against glucose-induced intracellular oxidative damage. Biochem. Biophys. Res. Commun..

[8] Guangdong Provincial Adverse Drug Reaction Monitoring Center (2004). Knowledge of adverse drug reactions (4) [J]. Guangdong Pharm..

[9] Lu PF, Xiang YY, Xie YM, Chang YP, Wang ZG (2013). Pharmacovigilance of parenterally administered salvianolate based on analysis of spontaneous reporting system data. Chin. J. Chin Mater. Med..

[10] Zhu YX, Sun XZ, Li CH (2015). Adverse reactions of salvia miltiorrhiza polyphenols for injection in 2 cases. J. North. Pharm..

[11] Zhang JP (2015). Efficacy of Oxiracetam combined with salvianolate in the treatment of senile cerebral infarction. China Med. Innov..

[12] Yan XQ (2018). Literature Analysis for Adverse Reactions of salvia miltiorrhiza polyphenols for injection from 2005 to 2016. World Latest Med. Inf..

[13] Wang HY (2016). Safety analysis of the effects of salvianolate injection on renal function in the real-world. Chin. Drug. Alert.

[14] Chang YP, Huo J, Xie YM, Zhang H, Zhuang Y (2013). Real-world study of affect on liver function of overdose of salvianolate extract injection. Chin. J. Chin Mater. Med..

[15] Lv SW, Guo JY, Zhu YL (2016). Investigation and analysis of clinical use and safety of salvia miltiorrhiza polyphenols for injection. Chin. J. Rural. Med. Pharm..

[16] Zhao Y, Wang T, Li G (2018). Pharmacovigilance in China: development and challenges.Int. J. Clin. Pharm..

[17] Li X (2018). Active pharmacovigilance in China: recent development and future perspectives. Eur. J. Clin. Pharmacol..

[18] Wang J, Zhang M, Li S (2018). Adapting and applying common methods used in pharmacovigilance to the environment: A possible starting point for the implementation of eco-pharmacovigilance. Env. Toxicol. Pharmacol..

[19] Tiberio López G (1992). Adverse drug reactions: Naranjo’s and Venulet’s algorithms. Rev. clínica española.

[20] Naranjo CA (1981). A method for estimating the probability of adverse drug reactions. Clin. Pharmacol. Ther..

[21] Higgins J. P. T, Altman D. G. & Sterne J. A. C. Chapter8:assessing risk of bias in includsd studies, cochrane handbook for systematic reviews of interventions version 5.1.0[EB/OL]. (2011-03-21). [2013-10-10]. http://handbook.cochrane.Org.

[22] Stroup DF (2000). Meta-analysis of observational studies in epidemiology: a proposal for reporting. JAMA.

[23] Wang C (2018). Re-evaluation of the post-marketing safety of Shuxuening injection based on real-world and evidence-based evaluations. Drug. Des. Devel Ther..

[24] Zhuang Y, Xie B, Weng S (2011). Construction and realization of real world integrated data warehouse from HIS on re-evaluation of post-maketing traditional Chinese medicine. Chin. J. Chin Mater. Med..

[25] Higgins JP, Thompson SG, Deeks JJ (2003). Measuring inconsistency in meta-analyses. BMJ.

[26] Kriston L (2013). Dealing with clinical heterogeneity in meta-analysis. Assumptions, methods, interpretation. Int. J. Methods Psychiatr. Res..

[27] Ym X, Mao P, Tian F (2010). Exploration of the Application Prospect of Real World Research in Post-marketing Clinical Re-evaluation of Traditional Chinese Medicine. Tradit. Chin. Drug. Res. Pharmacol..

[28] Wang C (2019). Re-evaluation of the post-marketing safety of Xuebijing injection based on real-world and evidence-based evaluations. Biomed. Pharmacother..

[29] Li C (2018). Post-marketing safety surveillance and re-evaluation of Xueshuantong injection. BMC Complement. Altern. Med..

[30] Li X. C, Dai G. H. & Liu X. C. Real World Study Methods and Its Application in Clinical Efficacy Evaluation of Traditional Chinese Medicine Based on HIS. *J Shandong Univ Tradit Chin Med* 415–418, 10.16294/j.cnki.1007-659x.2016.05.006 (2016).

[31] Ernster VL (1994). Nested case-control studies. Prev. Med..

[32] Mayrink J (2019). Incidence and risk factors for Preeclampsia in a cohort of healthy nulliparous pregnant women: a nested case-control study. Sci. Rep..

[33] Najafpour Z, Godarzi Z, Arab M (2019). Risk Factors for Falls in Hospital In-Patients: A Prospective Nested Case Control Study. Int. J. Health Policy Manag..

[34] Bezin J (2019). Use of Lipid-Lowering Drugs and the Risk of Cataract: A Population-Based Nested Case-Control Study. Clin. Pharmacol. Ther..

[35] Cao XF, Kong FF (2018). Analysis of 36 cases of adverse reactions of salvia miltiorrhiza polyphenols for injection. Chin. J. Clin. Ration. Drug. Use.

[36] Xu YQ (2016). Analysis of allergic reactions induced by salvia miltiorrhiza polyphenols for injection in 21 cases. China Health Stand. Manag..

[37] Yu YX, Wang C, Liu GH (2017). Literature review of adverse reactions of salvia miltiorrhiza polyphenols for injection. Chin. J. Drug. Abus. Prev. Treat..

[38] Chen ZW, Xie YM, Liao X, Wang GQ (2016). Systematic review on safety of salvianolate injection. Chin. J. Chin Mater. Med..

[39] Qiu YH, Yao C, Shen J, Kong FF, Guo LJ (2015). Observation on the Rationality and Safety of Clinical Application of salvia miltiorrhiza polyphenols for injection in Our Hospital. China Med. Her..

[40] Zhu YX, Li CH, Sun XZ, Sun RQ, Gong YP (2015). Observation and analysis of clinical adverse reactions of salvia miltiorrhiza polyphenols for injection. China Contin. Med. Educ..

[41] Lv SW, Guo JY, Zhu YL (2015). Practice of post-marketing safety re-evaluation of traditional Chinese medicine injections such as salvianolate and other drugs in our hospital. J. China Pharm..

[42] Liao X, Chang YP, Xie YM, Huo J, Zhang H (2014). Analysis of questionable allergic factors to parenterally administered salvianolate–a nested case control study using hospital information system data. Chin. J. Chin Mater. Med..

[43] Ren X, Xie N, Xu XY, Xuan LJ (2012). Study on the Compatibility Stability of salvia miltiorrhiza polyphenols for injection and 21 Commonly Used Clinical. Medicines. J. China Pharm..

