# Supplementary material for: Liver function monitoring: a prospective nested case-control study of Salvia miltiorrhiza polyphenol injection
Source: Sci Rep. 2020 Feb 26;10:3538. doi: 10.1038/s41598-020-60608-z (PMC7044424; doi:10.1038/s41598-020-60608-z)
Supplement: Supplementary file 1 — Dataset 1. [file 41598_2020_60608_MOESM1_ESM.pdf]

Liver function monitoring: a prospective nested case-control study of *Salvia miltiorrhiza* polyphenol injection

Jin-quan Cheng[1,2], Qing-ping Shi[1,2*], Feng Ding[1], Ling-ti Kong[1,2], Mei-ling Yu[1,2], Can Wang[2]

[1]Department of Pharmacy, the First Affiliated Hospital of Bengbu Medical College, Bengbu, Anhui, China

[2]School of Pharmacy, Bengbu Medical College, Bengbu, Anhui, China

[*]**Corresponding Author:** Qing-ping Shi: Department of Pharmacy, the First Affiliated Hospital of Bengbu Medical College, 287 Changhuai Road, Bengbu, 233004, Anhui, China. E-mail: sir_shi@126.com. Tel: +86 0552 308 6246

Supplementary Table 1. Variables and assignments included in the study

Variables	Case group(n=58)	Control group(n=232)	Classification variable assignment
**Sex**			
Male	31 (53.45%)	133 (57.33%)	0
Female	27 (46.55%)	99 (42.67%)	1
Age (years)	64.12±16.28	64.41±16.86	-
**Drug allergy history**			
Sulfa	2 (3.45%)	11 (4.74%)	Yes=1, No=0
Cephalosporins	1 (1.72%)	14 (6.03%)	Yes=1, No=0
Levofloxacin	2 ((3.45%)	0	Yes=1, No=0
Oxytetracycline	1 (1.72%)	1 (0.43%)	Yes=1, No=0
**Payment methods**			
Self-pay	18 (31.03%)	38 (16.38%)	0
Medical insurance	40 (68.97%)	194 (83.62%)	1
**Admission condition**			
Serious condition	20 (34.48%)	49 (21.12%)	0
General condition	38 (65.52%)	183 (78.88%)	1
**Dosing regimen**			
Single dose(mg)	173.28±45.69	1849.14±1376.92	-
Total dose(mg)	176.29±39.37	1580.60±1019.97	-
**Solvent**			
5% glucose injection	41 (70.69%)	168 (72.41%)	0
0.9% sodium chloride	15 (25.86%)	49 (21.12%)	1
Xylitol injection	2 (3.45%)	5 (2.16%)	2
Invert sugar electrolyte	0	8 (3.45%)	3
5% glucose sodium chloride	0	1 (0.43%)	4
Fructose sodium chloride	0	1 (0.43%)	5
Duration of administration (d)	10.21±6.93	10.46±7.74	-
**Comorbidities**			
Heart disease	9 (15.52%)	50 (21.55%)	Yes=1, No=0
Hypertension	7 (12.07%)	19 (8.19%)	Yes=1, No=0

Diabetes	13 (22.41%)	59 (25.43%)	Yes=1, No=0
Cerebral infarction	8 (13.79%)	49 (21.12%)	Yes=1, No=0
Kidney disease	7 (12.07%)	12 (5.17%)	Yes=1, No=0
Liver Disease	2 (3.45%)	10 (4.31%)	Yes=1, No=0
Smoking / drinking history	4 (6.90%)	27 (11.64%)	Yes=1, No=0
**Combined drugs (34 other drugs used in combination are summarized at the end of the table)**			
10% potassium chloride	29 (50%)	54 (23.28%)	Yes=1, No=0
Cefathiamidine	17 (29.31%)	31 (13.36%)	Yes=1, No=0
Torasemide	9 (15.52%)	16 (6.90%)	Yes=1, No=0
Levofloxacin	19 (32.76%)	52 (22.41%)	Yes=1, No=0
Pantoprazole sodium	19 (32.76%)	93 (40.09%)	Yes=1, No=0
Aspirin enteric-coated tablets	12 (20.69%)	78 (33.62%)	Yes=1, No=0
Low molecular heparin calcium	18 (31.03%)	43 (18.53%)	Yes=1, No=0
Human serum albumin	14 (24.14%)	16 (6.90%)	Yes=1, No=0
Dazhu Rhodiola injection	10 (17.24%)	17 (7.33%)	Yes=1, No=0

Ambroxol oral solution, Nikethamide injection, Piperacillin sodium and sulbactam, Rosuvastatin calcium tablets, Clopidogrel tablets, Metoclopramide injection, Furosemide injection, Aspirin-DL-lysine injection, Lansoprazole for injection, Ambroxol glucose, Dexamethasone injection, Terbutaline injection, Cefoperazone Sodium and Sulbactam Sodium, 10% sodium chloride, Lidocaine hydrochloride injection, Omeprazole sodium for injection, Granisetron glucose, Meropenem, Phenobarbitone, Cefotiam, Magnesium Sulfate injection, Penicillin sodium, Sodium aescinate, Reduced glutathione, Mannitol injection, 5% sodium bicarbonate, Cefdiazine sodium, Vitamin B6, Gentamicin injection, Heparin sodium injection, Low-Molecular-Weight Heparins sodium injection, Vitamin C, Doxofylline and Glucose Injection, Nitroglycerin injection

Note: Continuity data is expressed as mean + SD, and the classification data is expressed as the proportion of patients.
